# The USDA Barley Core Collection: Genetic Diversity, Population Structure, and Potential for Genome-Wide Association Studies

**DOI:** 10.1371/journal.pone.0094688

**Published:** 2014-04-14

**Authors:** María Muñoz-Amatriaín, Alfonso Cuesta-Marcos, Jeffrey B. Endelman, Jordi Comadran, John M. Bonman, Harold E. Bockelman, Shiaoman Chao, Joanne Russell, Robbie Waugh, Patrick M. Hayes, Gary J. Muehlbauer

**Affiliations:** 1 Department of Agronomy and Plant Genetics, University of Minnesota, St. Paul, Minnesota, United States of America; 2 Department of Crop and Soil Science, Oregon State University, Corvallis, Oregon, United States of America; 3 Department of Horticulture, University of Wisconsin, Madison, Wisconsin, United States of America; 4 The James Hutton Institute, Invergowrie, Dundee, United Kingdom; 5 USDA-ARS, Small Grains and Potato Germplasm Research Unit, Aberdeen, Idaho, United States of America; 6 USDA-ARS, Biosciences Research Lab, Fargo, North Dakota, United States of America; 7 Department of Plant Biology, University of Minnesota, St. Paul, Minnesota, United States of America; Oklahoma State University, United States of America

## Abstract

New sources of genetic diversity must be incorporated into plant breeding programs if they are to continue increasing grain yield and quality, and tolerance to abiotic and biotic stresses. Germplasm collections provide a source of genetic and phenotypic diversity, but characterization of these resources is required to increase their utility for breeding programs. We used a barley SNP iSelect platform with 7,842 SNPs to genotype 2,417 barley accessions sampled from the USDA National Small Grains Collection of 33,176 accessions. Most of the accessions in this core collection are categorized as landraces or cultivars/breeding lines and were obtained from more than 100 countries. Both STRUCTURE and principal component analysis identified five major subpopulations within the core collection, mainly differentiated by geographical origin and spike row number (an inflorescence architecture trait). Different patterns of linkage disequilibrium (LD) were found across the barley genome and many regions of high LD contained traits involved in domestication and breeding selection. The genotype data were used to define ‘mini-core’ sets of accessions capturing the majority of the allelic diversity present in the core collection. These ‘mini-core’ sets can be used for evaluating traits that are difficult or expensive to score. Genome-wide association studies (GWAS) of ‘hull cover’, ‘spike row number’, and ‘heading date’ demonstrate the utility of the core collection for locating genetic factors determining important phenotypes. The GWAS results were referenced to a new barley consensus map containing 5,665 SNPs. Our results demonstrate that GWAS and high-density SNP genotyping are effective tools for plant breeders interested in accessing genetic diversity in large germplasm collections.

## Introduction

Barley (*Hordeum vulgare* subsp. *vulgare*) was one of the first cereals that human communities of the Fertile Crescent domesticated about 10,000 years ago [1]. Barley played a key role in the establishment of the first Neolithic farming settlements and today is one of the world’s most important crops (FAOSTAT website. Available: http://faostat.fao.org). Barley is essential for the malting and brewing industries and it is an important animal feed. It also constitutes a staple food in several regions of the world due to adaptation to high altitudes, drought and soil salinity [2]. Renewed interest in food barley is derived from recent research confirming the health benefits of barley in human diets [3]–[5].

Meeting the increasing global demands for food in a time of climate change is agriculture’s greatest current challenge. Increased CO_2_ levels are predicted to decrease global crop yields as a consequence of overall higher temperatures. Higher temperatures, in turn, will trigger changes in precipitation, salinity, and both the occurrence and frequency of crop diseases and pest outbreaks [6], [7]. The genetic uniformity of current cultivars, due to decades of breeding with elite materials, may lead to greater vulnerability to the negative effects of climate change and it will also limit future genetic gains [8], [9]. A new generation of breeding strategies is focused on finding novel sources of genetic variation that can be incorporated into breeding programs and thus continue making gains in both productivity and quality while at the same time responding to climate change [7], [10].

Being one of the most widely adapted crops, the barley germplasm pool has the potential to contain enough genetic diversity to breed for adaptation to different environmental conditions. Moreover, the ample barley germplasm resources available worldwide [11], [12] likely contain beneficial allelic variation that new genomic and breeding technologies can exploit [13]. The characterization and use of barley germplasm arrays for identifying candidate genes via genome-wide association studies (GWAS) has shown promising results. For example, the use of elite US and UK breeding germplasm (BarleyCAP and AGOUEB, [14]) coupled with the development of high-throughput barley SNP assays [15] has allowed the detection of quantitative genetic factors for biotic and abiotic stress resistance [16]–[19], and agronomic and/or grain quality traits [16], [20], [21]. This same strategy can be used for isolating important genes, as evidenced by the cloning of *INTERMEDIUM-C* (*INT-C*), one of the genes controlling spike architecture in barley [22].

The USDA-ARS National Small Grains Collection (NSGC) is one of the largest collections of barley germplasm in the world [12], [23]. The NSGC is comprised of 33,176 barley accessions that have been acquired and maintained over the past 100 years. These include cultivars, breeding lines, landraces, and genetic stocks from more than 100 countries [11], [24]. A subset representing approximately 10% of the entire collection (the ‘NSGC Barley Core’) was established in 1995 – with final additions in 2006 – by randomly selecting accessions based on the logarithm of the total number of entries from each country of origin, with a minimum of one accession per country [11]. The Core (as it will be referred to in the remainder of this manuscript) has been, and is being evaluated for various agronomic, spike and seed morphology traits, as well as resistance to diseases and pests (see www.ars-grin.gov/npgs for a list of traits for which data are available). Reports on phenotypic diversity for feed quality traits [25] and resistance to four major barley diseases and the Russian wheat aphid (RWA) [26] have been published. Simple sequence repeat (SSR) and Diversity Array Technology (DArT) markers were used to genotype subsets of the Core in order to determine the probable origins of unknown accessions [24] and to identify genomic regions associated with RWA resistance [27]. A more thorough genetic characterization of the Core is required to fully exploit this diverse germplasm through association genetics.

The Triticeae Coordinated Agricultural Project (TCAP) is focused on genetically and phenotypically characterizing wheat and barley germplasm pools to identify valuable alleles that can be used to develop varieties better adapted to climate change-related stresses ([28]; The TriticeaeCAP website. Available: http://www.triticeaecap.org/. Accessed Jan 2014). As part of this USDA-NIFA funded project, 2,417 accessions belonging to the Core were genotyped with a barley iSelect SNP platform, the highest-density genotyping array currently available for barley [29]. We have used these genotype data to (i) identify redundant accessions, (ii) assess population structure, (iii) determine patterns of LD, and (iv) develop mini-core sets capturing the majority of the allelic diversity present in the Core. These subsets of accessions will be useful for identifying the genes determining phenotypes that are particularly expensive and/or difficult to measure. To validate the utility of the Core for identifying loci determining quantitative and qualitative traits, we conducted GWAS on hull cover, heading date, and spike morphology. The GWAS was anchored by a consensus genetic map based on merging twelve SNP-based genetic maps. This high-density map provides a superior framework for GWAS and the subsequent introgression of candidate genes/genomic regions via plant breeding.

## Results

### Genetic Characterization of the Core and Identification of Redundant Accessions

A complete genetic characterization of germplasm collections is necessary to make the diversity contained therein efficiently accessible to plant breeders. A barley iSelect Illumina SNP platform [29], which includes 7,842 SNP markers, was used to genotype 2,417 accessions belonging to the Core. After genotype calling, quality control (QC) filtering was applied for both SNPs and samples to remove low-quality markers and accessions that performed poorly in the SNP assay (see ‘Materials and Methods’ for more information). A total of 6,224 SNP markers and 2,298 barley accessions passed the QC criteria.

Genetic redundancy is a common problem for genebank curators. A common cause is the unwitting submission of the same accession, with different names/designators. Based on phenotype alone, it is not possible to identify redundant accessions and the maintenance of duplicated materials invokes unnecessary and costly efforts. High-throughput genotyping technologies can cost-effectively identify redundant accessions. Based on all pair-wise SNP calls, we detected 178 sets of two or more genetically identical accessions involving a total of 520 individuals. For subsequent analyses, one accession per set was retained, provided that the geographic and/or phenotypic information assigned to individuals within a set was consistent. Of the 520 accessions, 82 were retained and 438 were removed (Table S1).

The final set of accessions, henceforth referred as the informative Core (iCore) contains 1,860 unique accessions from 94 countries and is comprised of 815 landraces, 781 cultivars/breeding lines, 21 genetic stocks and 243 accessions of undefined improvement status (Table S2).

### iSelect Consensus Genetic Map Development

An integrated consensus linkage map is a necessary reference point for characterizing and using genetic diversity. In this study, the genetic map provided a resource to assess the distribution of LD and divergent selection as well as the coordinates for genes and QTL identified by GWAS. For subsequent and more comprehensive GWAS and isolation of candidate genes, a robust and high-resolution linkage map is an essential resource for alignment with the genome sequence. We took advantage of the 2,832 barley OPA SNPs [15] represented on the iSelect array to integrate the 11 genetic maps used in the development of the current barley consensus map [30] with the iSelect SNP map generated using the Morex x Barke (MB) mapping population [29].

As shown in Table 1, a total of 5,665 markers were mapped into 2,032 unique consensus positions (bins). The map spans 1,113 cM, a value very similar to the average length of the 12 maps (1,087 cM), and provides an average density of one marker bin every 0.55 cM. Relatively few ordering conflicts were present in this set of linkage maps, ranging from zero for chromosomes 6H and 7H to seven for chromosomes 2H and 4H (Table 1). This consensus map contains 465 non-iSelect SNPs that correspond to previously mapped SNP markers not included in the new iSelect platform. If only iSelect SNPs are considered, we were able to map 5,200 markers, which represent an increase of 1,233 iSelect SNPs over the previous MB map. Table S3 contains both versions of the iSelect consensus genetic map: iSelect markers only and all SNPs. The map and supporting data are available at the Triticeae Toolbox (T3) database (Available: http://triticeaetoolbox.org/barley. Accessed Jan 2014).

**Table 1:** Statistics of the iSelect consensus genetic map.

Chromosome	Map Length (cM)	#markers	#bins	#conflicts
1H	145	588	248	4
2H	181	978	352	7
3H	164	892	337	2
4H	130	586	230	7
5H	185	1101	313	4
6H	139	738	253	0
7H	169	782	299	0
Total	1113	5665	2032	24

### Population Structure of the iCore

**Figure 1:**
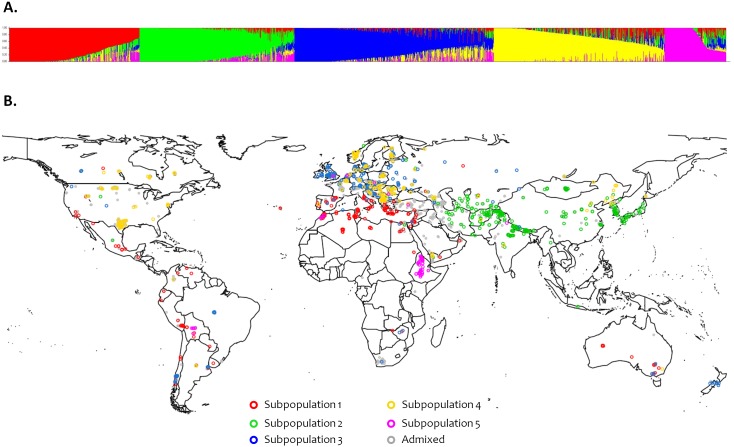
Population structure in the iCore. (A) Plot of Ancestry estimates for *k* = 5. Each bar represents the estimated membership coefficients for each accession in each of the five subpopulations (represented by different colors). (B) Geographical distribution of the accessions belonging to the iCore. A membership coefficient>0.8 was used to assign accessions (represented by circles) to the five subpopulations, and the remaining accessions were assigned to an ‘admixed’ group.

Population structure was evaluated using the software STRUCTURE v.2.3.4 [31] and by principal component analysis (PCA) using TASSEL v. 3.0 ([32]; Available: http://www.maizegenetics.net. Accessed Jan 2014). The estimated log probability of the data (LnP(D)) for each *k* between 1 and 10 increased continuously without reaching a plateau (Figure S1.A). To identify the optimal number of genetic clusters (subpopulations), Δ*k* values were also calculated as proposed by Evanno et al. [33]. The maximum Δ*k* value was reached at *k* = 2 (Figure S1.B), which mainly separates the two types of inflorescence morphology (‘spike row type’; two-row vs. six-row barley) and another lower peak was shown at *k* = 5. PCA was also performed on the dataset. As shown in Figure S2, the first principal component (PC1) mainly separates two-row from six-row barleys and the subsequent components (PC1–PC4) identified the same five subpopulations. Thus, both the STRUCTURE and PCA results indicate that there may be five subpopulations (*k*). Figure 1.A plots ancestry estimates for each accession in each of the five subpopulations. A membership coefficient >0.8 was used to assign accessions to subpopulations. Accessions within a subpopulation with membership coefficients ≤0.8 were considered ‘admixed’. All accessions were subsequently plotted according to their region of origin (Figure 1.B). If latitude and longitude data were not available in the Germplasm Resources Information Network (GRIN) system (Available: www.ars-grin.gov/npgs. Accessed Jan 2014), we used the geographical centers of the respective country or state/province as the geographic coordinates.

Most accessions within subpopulation 1 exhibit six-row spike morphology and traced to the Mediterranean countries, Australia, and regions of Central and South America (Figure 1.B; Table S2). Over half of the 200 individuals belonging to this subpopulation (115 accessions) are categorized as landraces (Table 2). Subpopulation 2 (273 accessions) is composed primarily of six-row Asian landraces, while most accessions in subpopulation 3 (274 accessions) are two-row cultivars/breeding lines from European countries (Figure 1.B; Table 2; Table S2). Cultivars from New Zealand, Brazil, Canada (Alberta), and some Chilean landraces also belong to this subpopulation. Subpopulation 4 contains 207 accessions – primarily six-row cultivars/breeding lines from Europe, the USA, and Canada. Macedonian and some Asian landraces are also included in subpopulation 4. Subpopulation 5 contains 86 accessions (both two-row and six-row), mainly Eritrean and Ethiopian landraces with a few from Morocco and Bolivia (Figure 1.B; Table 2; Table S2). Half of the iCore accessions (820) are ‘admixed’ and this ‘admixed’ cluster includes even numbers of landraces and cultivars/breeding lines (Table 2). Admixed landraces generally traced to the Middle East and the Caucasus region (Figure 1.B; Table S2).

**Table 2:** Composition of the genetic clusters defined by STRUCTURE.

Genetic cluster	#Landraces	#Cultivars & breeding lines	#Genetic stocks	#Undefined accessions	Total
Subpopulation 1	115	29	0	56	200
Subpopulation 2	199	40	0	34	273
Subpopulation 3	32	220	3	19	274
Subpopulation 4	47	135	7	18	207
Subpopulation 5	73	6	0	7	86
Admixed	349	351	11	109	820

**Figure 2:**
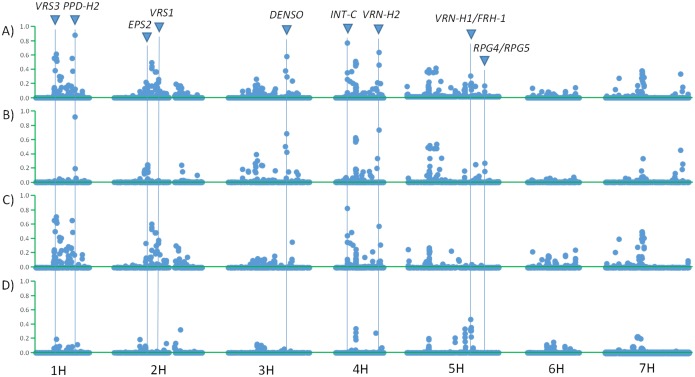
Genetic differentiation between subpopulations 2, 3 and 4. (A) Genetic differentiation measured by *Φ*_PT_ for subpopulations 2, 3 and 4 (A). To identify which subpopulation is responsible for the high values of some markers, we run independent analyses of divergent selection for: (B) subpopulation 2 against subpopulations 3 and 4; (C) subpopulation 3 against subpopulations 2 and 4; and (D) subpopulation 4 against subpopulations 2 and 3. To help discriminate markers with higher values, the Y-axis displays *Φ*_PT_ to the power of 10.

Subpopulation genetic differentiation, a tool for revealing the effects of selection, provides a complementary approach to understanding the main drivers of genetic differentiation in a germplasm array. It reveals genomic regions, or loci, at which the frequency of a certain allele in a subpopulation is significantly different than in the others. We applied this analysis to subpopulations 2, 3 and 4 because, based on the PCA, they are the most genetically distinct (Figure S2). These subpopulations also have similar numbers of individuals (Table 2). We found that many differentially selected genomic regions are coincident with, or near to, known loci involved in flowering time and spike row number (Figure 2). Specifically, we found evidence for genetic differentiation coincident with: the photoperiod gene *PPD-H2* [34] and the *earliness per se* locus *EPS2* [29] between subpopulation 2 and subpopulations 3 and 4; the vernalization gene *VRN-H1* [35] between subpopulation 4 and subpopulations 2 and 3; and the three main genes controlling spike row number *VRS1* [36], *VRS3* [37] and *INT-C* [22] between subpopulation 3 and subpopulations 2 and 4. Also, between subpopulation 2 and subpopulations 3–4 there is evidence for genetic differentiation in the vicinity of *RPG4/RPG5* (Figure 2), a complex locus involved in resistance to stem rust [38].

In the remainder of this report, we will use the following descriptors for significant associations: *within* means the SNP is within the determinant gene; *coincident* means the SNP and the known gene are in the same bin; *near* means within a few centi Morgans (cM). Further experiments are necessary to prove that a significant association is causal.

### Linkage Disequilibrium

**Figure 3:**
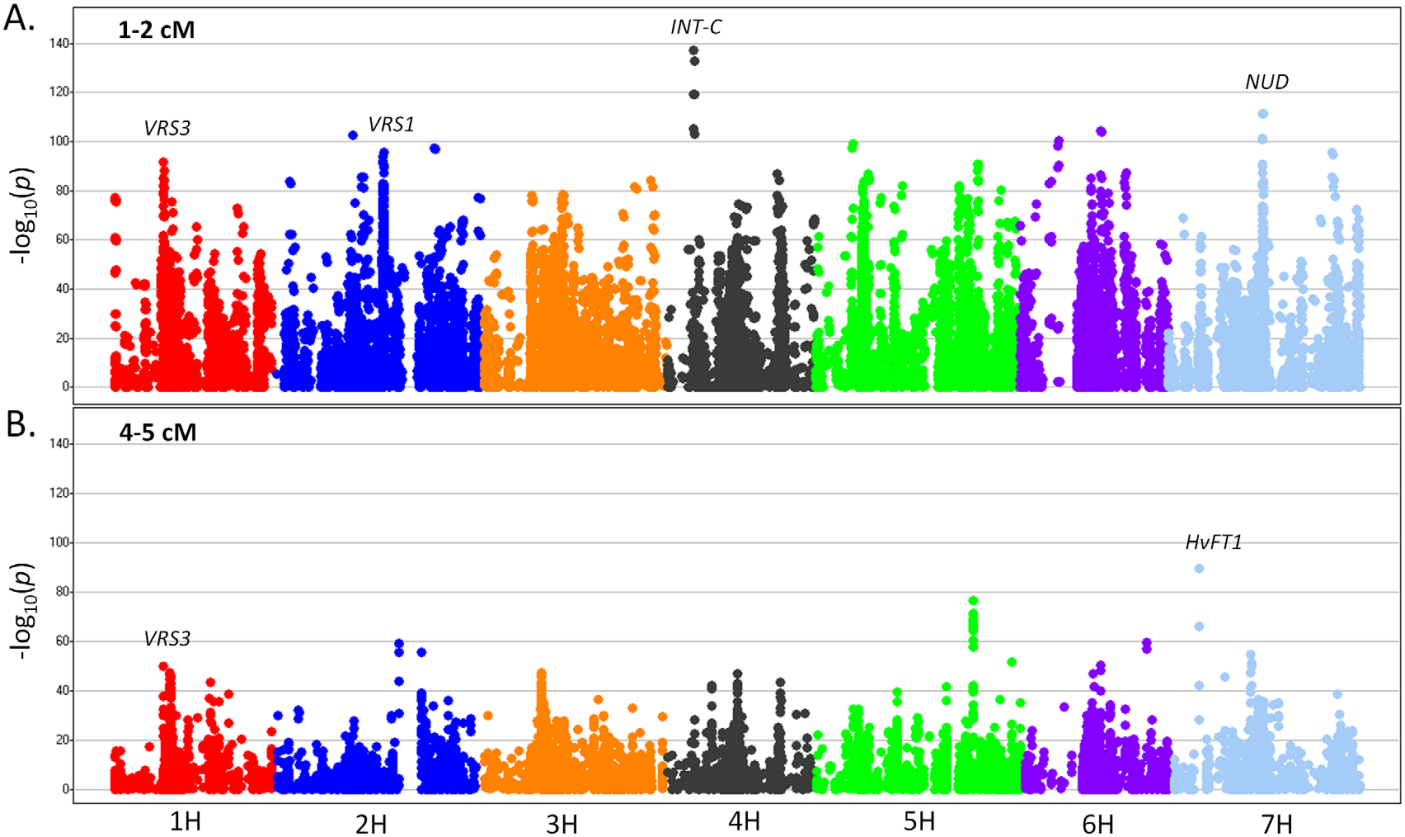
Distribution and extent of linkage disequilibrium along the barley chromosomes. The –log_10_ of the logistic regression *p*-values between any pair of SNPs located 1–2 cM apart (A) and 4–5 cM apart (B) are displayed.

The distribution and extent of LD was analyzed for the whole iCore. To account for population structure, we followed a logistic regression approach using principal components as covariates. The *p*-value of the logistic regression of any given marker with another marker located at a specified distance will be, therefore, a direct measurement of LD between the two markers without the confounding effect of population structure. We analyzed the *p*-value of the logistic regression between any pair of SNPs located 1–2 cM and 4–5 cM apart. In this manner, we systematically scanned along the chromosomes and displayed the extent and distribution of LD in two inter-marker distance intervals. Analyses of LD patterns in the genome can provide insights into recombination hotspots (very low LD) and selective sweeps (high LD) [39]. As shown in Figure 3, the LD pattern varies across the barley genome. Regions of high LD were found near *VRS3* on 1H, *VRS1* on 2H, *INT-C* on 4H and *NUD* on 7H at the 1–2 cM marker distance (Figure 3.A). The *VRS3* region still showed a high LD over longer genetic distances (4 to 5 cM; Figure 3.B). In some regions of the genome (e.g. near *HvFT1* on 7H and at 142 cM on 5H), high LD was detected at 4–5 cM distance but not at 1–2 cM (Figure 3.B). A reason for this could be a lack of mapped markers located 1–2 cM apart.

The rate at which LD (*r* ^2^) decays with genetic distance was investigated for each of the five subpopulations. The patterns of LD decay differ between subpopulations (Figure S3). LD decays faster in subpopulations 1 and 3 and is followed by a plateau of *r^2^* values. In subpopulations 2 and 4, however, there is a steadier rate of decay with genetic distance. With the exception of subpopulations 1 and 3, significance thresholds are different for each subpopulation. Threshold values are similar between chromosomes from all subpopulation but subpopulation 5, which shows a different significance threshold for each chromosome (Figure S3). The LD plots also reveal specific regions in high LD that are far apart. For instance, subpopulation 4 shows high LD between two regions of chromosome 4H separated by approximately 70 cM, which is not present in any of the other subpopulations.

### Genome-wide Association Studies (GWAS)

We performed GWAS to test if the natural diversity present in the iCore could be exploited to find genes via association genetics. We chose three phenotypes: ‘hull cover’, ‘spike row number’ and ‘heading date’, which range from monogenic and qualitative (‘hull cover’) to polygenic and quantitative (‘heading date’). ‘Hull cover’ refers to adherence, or lack thereof, of the hull (lemma and palea) to the seed and is controlled by a single locus (*NUD*) on chromosome 7HL [40]. ‘Spike row number’ refers to the number of fertile spikelets per rachis node of the inflorescence. The two-row vs. six-row phenotype is determined mainly by *VRS1* on 2H [36] but also by *INT-C* on 4H [22], *VRS3* on 1H [37] and a number of loci that modify *Vrs1* [41]. Heading date (or days to flowering), is a key trait for the adaptation of barley to different growing environments and it is controlled by many QTL associated with vernalization requirement, photoperiod sensitivity, and earliness *per se* [42]. Phenotypic data used for GWAS analyses were obtained primarily from evaluations of the Core available at GRIN (Germplasm Resources Information Network system. Available: www.ars-grin.gov/npgs. Accessed Jan 2014) and are shown in Table S2. As described in the Materials and Methods, we also use ‘spike row number’ and ‘heading date’ data from field trials conducted at Corvallis, Oregon, USA. All association results from GWAS are shown in Table S4.

**Figure 4:**
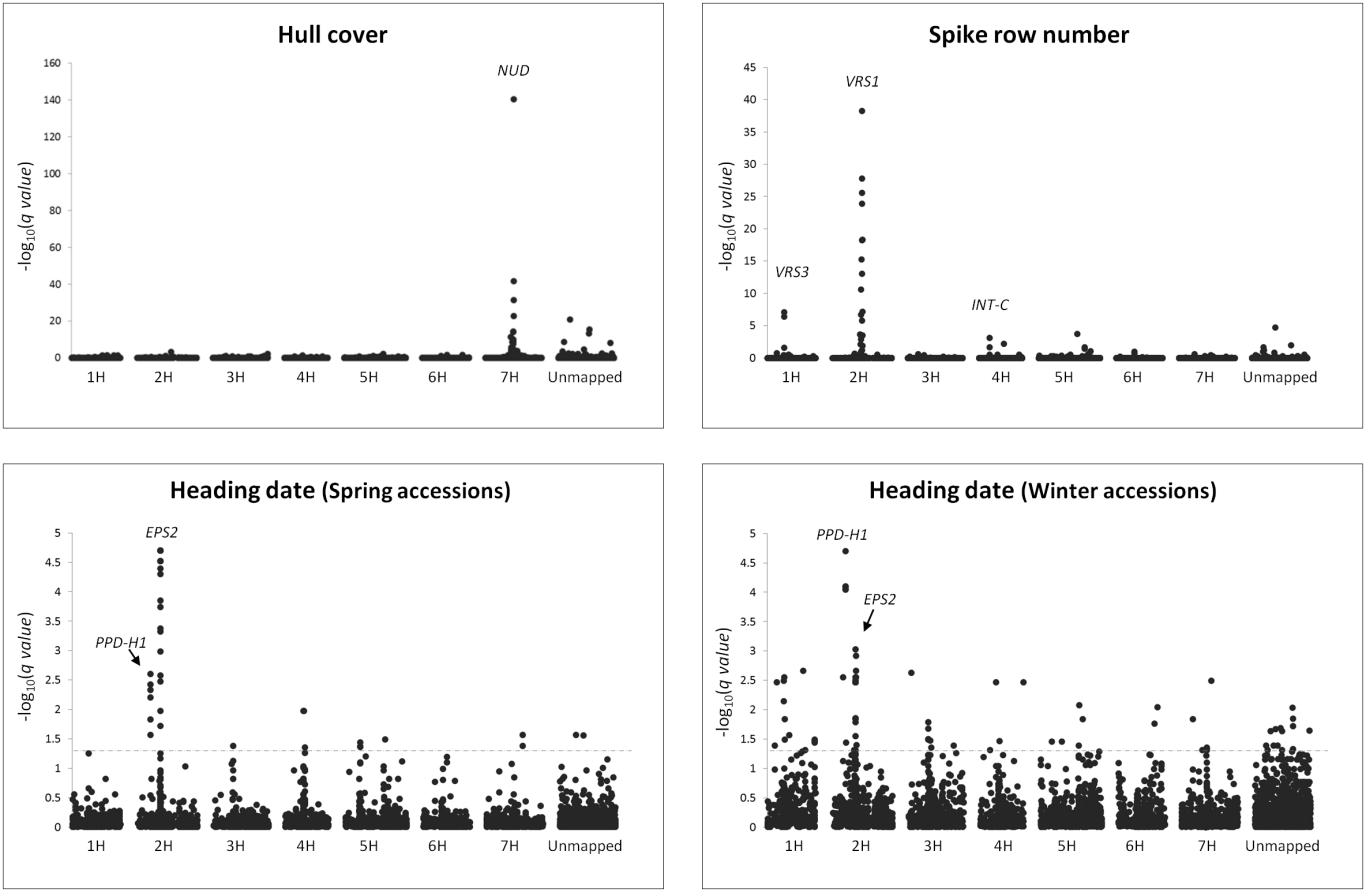
Genome-wide association scans in the iCore. Manhattan plots of the GWAS for ‘hull cover’, ‘spike row number’, ‘heading date’ in the spring accessions, and ‘heading date’ in the winter accessions are shown. The horizontal axes indicate the consensus map position of each SNP (black dots), while the vertical axes indicate the −log_10_ of the corrected *p* values (*q*). The dash line indicates the 0.05 threshold.

GWAS of ‘hull cover’ found highly significant SNPs (max –log_10_(*q*)  = 140.49) with the most significant marker located at 85.87 cM on chromosome 7H (Table 3; Figure 4). These significant SNPs are associated with the *NUD* locus that maps near SNP 12_30301 (85.87 cM; Table S3) in the Oregon Wolfe Barley (OWB) population [43]. A causative SNP in the gene cannot be tested with the iSelect array since only hulled barleys were used for SNP discovery.

**Table 3:** Significant SNPs showing the highest marker-trait associations for the phenotypes tested.

Trait	SNP	Chr.	Position (cM)	–log_10_(*q*)	Effect	MAF
Hull cover	12_20685	7H	85.87	140.49	–4.21 (A)	0.10
Spike row number	12_30896	2H	91.09	38.27	0.98 (A)	0.45
	11_10933	1H	51.06	7.08	0.71 (G)	0.31
	11_20606	4H	31.14	3.11	0.46 (G)	0.34
Heading date (spring lines)	BK_14	2H	38.6	2.60	3.08 (G)	0.44
	SCRI_RS_222769	2H	69.55	4.72	4.01 (A)	0.43
Heading date (winter lines)	12_30871	2H	38.6	4.70	–10.02 (G)	0.44
	SCRI_RS_127347	2H	69.55	3.03	7.87 (A)	0.49

The –log_10_ of the FDR corrected *p-*values (*q*) for those markers are shown, together with the allele effects (allele in parenthesis) and the minor allele frequency (MAF) for each marker.

We also identified significant SNPs in, or in high LD with, the three major genes determining barley ‘spike row number’. The top hit corresponds to SNP 12_30896 (91.09 cM on 2H; Table 3), which is located on a sequenced BAC containing the causative homeodomain-leucine zipper transcription factor gene for *VRS1* ([36], [44], [45]; HarvEST: Utilities Menu. Available: http://harvest-web.org/utilmenu.wc. Accessed Jan 2014). SNP 11_20606, at 31.14 cM on 4H (Table 3), is in high LD with *INT-C* and was one of the GWAS hits that allowed the identification and cloning of this gene [22]. Finally, marker 11_10933 (51.06 cM on 1H; Table 3) maps close to *VRS3* [37].

Heading date is an important trait in terms of yield and adaptation. We performed two independent experiments to measure heading date: one with only spring lines planted in the fall and another with the winter lines planted in mid-winter (see Materials and Methods for details). In both GWAS, the SNPs showing the highest significant associations were located on chromosome 2H, BK_14 and 12_30871 within *PPD-H1* [46] and SCRI_RS_222769 and SCRI_RS_127347 coincident with *EPS2* [29] (Figure 4; Table 3).

### Mini-core Sets

**Figure 5:**
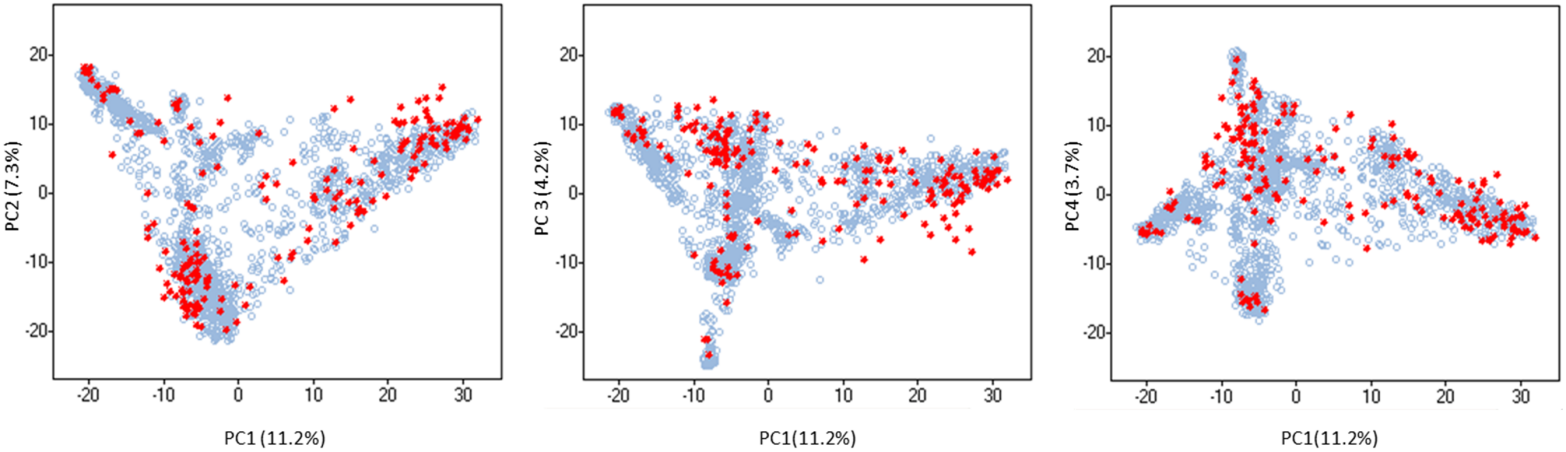
Principal Component Analysis (PCA) of the iCore and distribution of the ‘mini-core’ set in the first 4 PCs. The ‘mini-core’ set is shown in red and it is composed of the first 10% top-ranked accessions by their contribution to the polymporphism information content (PIC) value of the whole iCore.

To create sub-sets of accessions that maximize allelic diversity with the fewest possible numbers of accessions, we sorted all the iCore accessions by their contribution to the average polymorphism information content (PIC), based on 4,558 mapped SNPs. In a step-wise process we then identified, one at time, the accessions whose removal from the whole set led to the highest average PIC. If a set of *n* individuals shows the same or larger average PIC value when one individual was excluded, that indicates the excluded individual was not contributing to the diversity of the whole set and its presence was redundant in terms of diversity. The process was repeated with a set of *n*-1 individuals and another accession was excluded. Following this procedure, a point was reached where the removal of any other accession from the set decreased the average PIC. This means that the excluded line was contributing to the diversity of the whole set. With the current dataset, we reached this point with 37 accessions (Table S5). Choosing the accessions by their rank position in contribution to the PIC value ensures that the selected subset provides an informative sample of the allelic diversity in the entire collection (Table S5). As shown in Figure 5, a sample of the 186 top-ranked accessions (10% of the iCore) creates a mini-core comprised of 10 accessions from subpopulation 1; 14 accessions from subpopulation 2; 48 accessions from subpopulation 3; 23 accessions from subpopulation 4; 5 accessions from subpopulation 5; and 86 admixed accessions. The proportion of individuals taken from each population was not equal, which indicates different degrees of allelic diversity across populations. The overrepresentation of samples from subpopulation 3, which were mostly two-row European cultivars/breeding lines, was probably due to the ascertainment bias in the SNP array caused by the preferred use of these types of breeding materials for SNP discovery [29], [47].

We also ranked the accessions by contribution to the average PIC value within the spring two-row (n = 621) and spring six-row (n = 862) accessions (Table S5). We did not rank the winter 2-row and winter 6-row group because these groups consist of less than 200 accessions. The average PIC values for any subset from the two spring growth habit spike morphology groups are lower than those of subsets of the same size taken from the iCore, and some accessions that are top-ranked within the spring 2-row or the spring 6-row subsets are bottom-ranked in the iCore. These results indicate that these accessions are an important source of diversity within their groups but not in the iCore, where their genetic background is already represented.

## Discussion

### Genotypic Characterization Makes Germplasm Collections More Useful: the USDA-NSGC Model

Greater genetic diversity than is present in current elite crop varieties will be needed to meet future production goals and the challenges of climate change [7]. Fortunately, seeds of historical breeding materials, locally adapted landraces and/or wild relatives stored in germplasm collections constitute an extensive reservoir of biodiversity from which cultivated gene pools can be enriched. Although the ease of mobilization of favorable alleles into breeding materials is inversely related to the degree of adaptation, advances in genomics and molecular breeding technologies are able to accelerate the use of exotic germplasm for crop improvement [10], [28]. However, accessing novel genetic variation in genebank collections will require thoughtful and renewed characterization at the genotypic and phenotypic levels [48], [49].

The USDA-NSGC, the second largest germplasm collection of barley in the world (the largest is located at Plant Gene Resources of Canada in Saskatchewan; [12]), is an underused treasure of diversity. A core collection of 2,574 accessions was created within the NSGC to facilitate access to the diversity contained in the whole collection. Due to the lack of genotypic information and good morphological descriptors at that time, the Core was developed based on geographic source information with the goal of effectively sampling the genetic diversity in the full collection. To access the diversity within the Core, we used the latest SNP-based platform developed for barley [29] to genotype 2,417 barley accessions belonging to the Core.

The curated set of SNPs was first used to address one of the main problems of germplasm collections: redundancy. It is estimated that, worldwide, only one third of the total number of accessions conserved *ex situ* are distinct [50], and duplications occur within and between genebanks of the same crop. Maintaining redundant materials consumes a significant amount of genebank resources, but the identification of these duplicates has been neither cost-effective nor reliable until the arrival of the high-throughput genotyping and sequencing technologies. Over 14% of the accessions in the Core are redundant based on information from 6,224 informative SNPs. In almost half of the cases, redundant accessions have the same origin/passport information. However, there are as many cases of identical accessions coming from different geographic regions, having different phenotypic data, and/or genetically identical accessions having different names (Table S1). Duplicates can be handled in different ways including: (1) keeping one accession and permanently removing the rest, (2) combining the seeds of duplicated accessions, or (3) removing identical accessions only from the ‘active’ Core [49]. For the purposes of our subsequent analyses, we retained one accession per set of duplicates with equal passport data and we refer to this non-redundant and non-ambiguous germplasm array as the iCore.

### The Five Subpopulations in the iCore Correspond to Principal Germplasm Groups

The principal determinants of population structure within the iCore are ‘spike row number’ and geographical origin. The ancestral condition of barley is two-row; the recessive six-row form was selected shortly after domestication [36]. Subsequently, there has been geographic separation of the two spike morphologies, and this separation has been reinforced by modern plant breeding due to the general practice of breeders not making crosses between the two germplasm groups. A second major division is growth habit: spring and winter forms are most adapted to, and widely grown in, different regions. Spike row number, growth habit, and origin are usually the principal sources of structure/classification in diverse arrays of barley germplasm (e.g. [29], [51]–[53]).

We identified five subpopulations within the iCore and all but subpopulation 5 were principally two-row or 6-row. Subpopulation 5, which consisted primarily of Eritrean and Ethiopian landraces, was quite distinct from other African and Asian accessions and included both two-row and six-row types and an intermediate type of labile barley, exclusive to this part of Africa, whose main feature is a different number of grains at each rachis node [54], [55]. The genetic distinctiveness of barley germplasm from the Horn of Africa has been reported previously [56], [57]. Some accessions from Morocco and all the accessions from Bolivia were included in subpopulation 5. It is likely that this genetic relatedness is due to introduction rather than convergent evolution, as Ethiopian and Eritrean landraces have been widely used in breeding programs as excellent sources of resistance to biotic and abiotic stresses [58]. Most accessions from Mediterranean countries belong to subpopulation 1 and are six-rows. In general, germplasm from this region is distinct from Central and Northern European accessions in terms of adaptation to the mild winters and hot summers that are characteristic of the Mediterranean climate [59]. Many accessions from Central and South America, as well as Mexico and California, belong to this subpopulation. Cultivated barley was introduced to the Americas by the Spanish nearly 500 years ago and the similarity in climate and continual human migration has likely led to subsequent introductions and exchanges [60].

Although the five subpopulations, in general, correspond to known germplasm groups, care must be taken when considering this collection as a representative sample of barley geographical diversity (present or past) or as a tool to explain origin, domestication or breeding history. There are several reasons why caution is prudent, including: (1) accessions were not always collected *in situ* (many were obtained from other collections and the collection location was recorded as geographic origin of the accession); (2) accessions may have been chosen based on diversity rather than on representing the principal germplasm group(s) grown at that location; (3) accessions described as landraces may actually be admixed cultivars; and (4) incorrect passport information. Examples of bias in the iCore include, but are not restricted to, the absence of two-row accessions from Australia, where two-row varieties prevail, and Manchurian types from the upper Midwest of the USA, which were the foundational varieties and have been extensively used in breeding programs [61]. There is also overrepresentation of lines coming from Texas (USA). Texas is not a principal barley growing area of the US: the germplasm was donated upon closure of a breeding program. As more accessions from the NSGC and other barley germplasm collections are genotyped, it may be feasible to incorporate new materials into the NSGC Core collection to better reflect patterns of germplasm distribution and diversity.

### High LD in the iCore is found in Genomic Regions that Contain Traits Involved in Domestication and Breeding Selection

Examination of linkage disequilibrium in cultivated barley has been the subject of numerous studies (e.g., [51], [62], [63]). To date, most of these studies have involved a limited number of individuals in highly structured collections. LD is a measure that has to be taken cautiously because of its variability across genetic backgrounds and is therefore highly population-specific, and the fact that since LD varies across the genome, it is usually considered in terms of average values. Nonetheless, LD patterns can be a useful tool for understanding recombination, breeding and selection history. LD also has implications for the resolution of GWAS at any given marker density and significant LD values between physically unlinked markers may give an idea of the number of false positives.

When we plotted the *p*-value of a logistic regression between pairs of markers located at a certain distance (between 1–2 cM and 4–5 cM apart), we identified varying degrees of LD across the genome. The fixation of natural mutations by selection can have a large impact on the patterns of LD in nearby regions [39]. Positive alleles at important loci would be fixed during domestication and breeding, and it is thus expected that regions with low marker density (lack of polymorphism) surrounded by regions with high LD would be diagnostic of selective sweeps. We found genomic regions that may be evidence of selective sweeps - important for all accessions regardless of subpopulation membership, geographic origin, or morphological attributes - for traits defining cultivated barley: *VRS3* on 1H, *VRS1* on 2H, *INT-C* on 4H and *NUD* on 7H (Figure 3.A). The differential selection at these and other loci involved in flowering time and disease resistance between subpopulations in the iCore can be responsible of the high *Φ*_PT_ values for SNPs located in those genome regions (Figure 2).

LD varies between subpopulations. The fastest decay occurs in subpopulations 1 and 3. While most accessions in subpopulation 1 are landraces, subpopulation 3 contains mainly cultivars and breeding lines (Table 2). It is generally understood that cultivated barleys have higher LD than landraces and wild barleys [64]. However, breeding strategies also vary and, although most breeders tend to cross closely related germplasm [65], [66], many times breeding involves more purposeful crossing with exotic materials [67]. Due to the wide geographical distribution of accessions within subpopulations, the lack of knowledge about the breeding strategies involved in their development and the fact that they are not true natural populations, it is difficult to draw any conclusion from the LD found in each iCore subpopulation.

### GWAS Identifies Genes Determining Traits of Varying Complexity

High-throughput genotype data coupled with phenotypic data in diverse germplasm arrays can be used to identify marker-trait associations via GWAS [68]. The more diverse the germplasm, however, the more important it is to account for structure to reduce the false discovery rate [69]. Statistical methods are constantly being improved to provide accurate predictions (e.g. [70], [71]). Given these considerations, we chose phenotypic data varying in their complexity of inheritance to assess the utility of GWAS in the iCore. Some of the genes determining these traits have been cloned, providing an opportunity for validation of significant marker-trait associations identified in GWAS. A number of recent advances in barley genomics (reviewed in [28]) facilitate the gene discovery process. Starting with significant SNP associations in GWAS one can, in many cases, efficiently identify one or more candidate genes for the target trait.

The presence or absence of adhering hulls is a simply inherited trait (‘hull cover’) determined by the *NUD* locus on chromosome 7HL that encodes an ethylene response factor (ERF) family transcription factor [40]. Since the *nud* allele is only present in subpopulation 2 (principally composed of accessions from Asia, where the hull-less trait is associated with higher levels of human food use than elsewhere in the world), the mixed linear model properly removed false associations due to structure and we identified significant marker-trait associations at the corresponding chromosome region (Figure 4). Although GWAS identified the genomic region where the *NUD* locus is, if the *NUD* gene had not been previously reported identifying it based on GWAS and the hulled/hull-less phenotype data would have been challenging due to the a number of factors including: (1) the lack of causative SNP(s) because of the ascertainment bias in the iSelect SNP data; and (2) the poor map resolution due to extensive LD found on chromosome 7H in the vicinity of *NUD* (Figure 3). In the case of ‘spike row number’, where at least three determinant genes are reported in the literature (*VRS1*, *INT-C*, and *VRS3*), GWAS narrowed down gene targets for cloned genes to a BAC clone (*VRS1*) and tight linkage (*INT-C*). The third gene, *VRS3,* has not been identified yet but mapping data confirms that it is located near the region identified by our GWAS (Waugh R., unpublished data).

‘Hull adhesion’ and ‘spike row type’ are qualitative traits and the effectiveness of GWAS was validated in that it correctly identified chromosomal regions previously reported to contain loci and/or genes determining each trait. In the case of the ‘hull adhesion’ trait, the two phenotypes are quite discrete and easy to score (e.g. the caryopsis has an adhering hull or it doesn’t). The GRIN data for this trait are binary. There are, however, quantitative differences in the degree of hull adherence within the adhering hull class [72] and the significant SNPs associated with ‘hull adhesion’ on regions other than the *NUD* locus are therefore possible candidates for this quantitative variation. ‘Spike row type’ is somewhat more challenging to phenotype than ‘hull adherence’, as there are intermediate types as a result of different *VRS1* alleles (labile barleys, [55]) and interactions with up to ten other *intermedium* (*int*) loci distributed across the genome [41].

Heading date, a surrogate measurement of flowering time, is a critical trait in terms of barley adaptation and shows the most complex inheritance of the three traits we studied. At least 6 major genes/QTL are reported to determine heading date in the literature [73], [74] as well as numerous small-effect QTL distributed across the whole genome. At the same time, heading date is relatively straightforward to phenotype under field and greenhouse conditions and therefore has a high heritability [75]. In this study, heading date was phenotyped separately for the ‘spring’ and ‘winter’ growth habit accessions in the NSGC core due to seed availability constraints. The latter were fall-sown (2011) and the former sown in mid-winter (2013) and thus the results are somewhat confounded by photoperiod duration and temperature. We performed separate GWAS analyses for the two groups of accessions.

The classification of growth habit was made by NSGC curators based on phenotype (vernalization sensitivity under non-vernalizing conditions) (Table S2). In our analysis, members of the ‘spring’ sets are found in all the subpopulations. The ‘winter’ accessions belong mostly to subpopulation 2 (37% of the accessions in this subpopulation are winter), subpopulation 4 (19%) and the admixed group (18%). Very few winter accessions are present in subpopulation 1 (6%), subpopulation 3 (1%) and none in subpopulation 5. GWAS of both datasets identified *PPD-H1* (SNP within the causative gene), a pseudo-response regulator (*PRR7*) involved in flowering time under long day conditions [46]. This gene is a key determinant of adaptation because the insensitive allele prolongs flowering under long-day conditions, thus maximizing yield potential. The GWAS also identified SNPs in tight linkage with *EPS2*, a homolog of *Antirrhinum CENTRORADIALIS* which is a main determinant of adaptation in spring barleys [29]. GWAS of ‘heading date’ in the winter accessions also identified this gene (Figure 4). Interestingly, we found evidence for differential selection at this locus in subpopulation 2 (Figure 2). As expected given the fall and winter planting dates ensuring ample opportunities for vernalization, there were no associations with the major vernalization genes *VRN-H1* or *VRN-H2*.

### Mini-core Sets Effectively Sample Genetic Diversity

Even after removal of redundant accessions, the collection is too large for most breeding programs to sample in a cost-effective fashion. We developed objective criteria, based on SNP diversity, for sampling the full collection to create mini-core sets, a procedure that will be of utility to all who seek to efficiently perform phenotyping of germplasm collections [76]. Our method, which is based on calculating the PIC value for each marker and the average for the whole set of markers, is a progression from structured random sampling, which involves dividing the whole collection into groups based on morphological, ecological or geographical criteria and then selecting a weighted number of individuals within each group [77]. The optimum population size is a question that any user of a collection faces. Once a user determines how many lines can be effectively phenotyped, this number can be selected from the iCore based on the rank. It is not recommended to select a number of lines smaller than the rank when the PIC reaches its maximum (37 when considering the whole iCore). However, practical use of a subset for GWAS will require more than this minimum number of accessions. We have also shown that *a priori* selection of a subset of accessions from the whole collection based on a certain criteria (e.g. ‘spike row type’) will lead to different mini-core sets of accessions. It is worth mentioning that, when selecting a ‘mini core set’, researchers should be aware of the ascertainment bias in the iSelect SNP array, which will lead to a higher representation of the breeding materials than landraces. Information regarding subpopulation membership coefficients, geographical origin and phenotypic data provided for each accession (Table S5) can help choosing sets depending on the purpose of the mini-core collection. In the future, either a deeper SNP discovery panel or a genotyping by sequencing (GBS) approach [78] should be used to accurately estimate diversity in more diverse sets of germplasm.

In summary, we have shown that the iCore is a highly diverse collection of barley genetic resources whose effective use will be maximized due to the availability of high density SNP data. The SNP data provide objective criteria for removal of redundant accessions and, as needed, for subsampling ‘mini-core’ sets of accessions for more efficient, cost-effective, or in-depth phenotyping. The high-throughput genotyping data – coupled with a newly developed high-density genetic map – were used to assess patterns of population structure and linkage disequilibrium that we applied to gene discovery using GWAS. For each of three model traits, GWAS identified significant marker-trait associations. The SNPs involved in these associations were in genes known to be responsible for the phenotype, physically linked to determinant genes, and tightly linked to determinant genes/loci. Genotypic and phenotypic data, together with the iSelect consensus map, have been uploaded to T3 (The Triticeae Toolbox website. Available: http://triticeaetoolbox.org/barley/. Accessed Jan 2014) to accelerate the utilization of the genetic diversity contained within the USDA’s remarkable collection of barley accessions.

## Materials and Methods

### SNP Genotyping and Data Curation

A total of 2,417 barley accessions belonging to the USDA-NSGC Barley Core were genotyped using the Infinium iSelect SNP assay according to the manufacturer’s protocol (Illumina Inc., San Diego, CA, USA). The whole core collection contains 2,574 accessions but only 2,417 were genotyped. Automated SNP calling was performed using the cluster algorithm implemented in GenomeStudio v.2011.1 software (Illumina Inc., San Diego, CA, USA). SNP calls were manually inspected to verify their accuracy. Briefly, genotype clusters were manually adjusted for those SNPs with inaccurate cluster definitions, and SNPs producing theta-compressed clusters were excluded. Heterozygous SNP calls were converted to missing values. Standard QC filters were applied to the resultant dataset to remove low-quality SNPs and samples: SNPs with missing calls in >10% accessions as well as accessions with missing calls in >10% of the SNPs were removed from further analysis.

The curated SNP dataset consisting of 6,224 SNP markers was used to identify potential duplicates in the NSGC barley core. A pairwise similarity matrix based on simple matching coefficients was built and accessions sharing all alleles were exported. Those genetically identical accessions were also confirmed by DArT markers previously scored in the collection (JM Bonman, personal communication). Table S1 contains the list of redundant accessions found in the collection.

### Development of the iSelect Consensus Map

The 11 linkage maps used by Muñoz-Amatriaín et al. [30] and the iSelect MB map generated by Comadran et al. [29] were integrated using LPmerge ([79]). This software has two parameters: one is the maximum interval size between bins to include in the objective function, which was varied between 1 and 4; the second is the weights to apply to each map. For each maximum interval, a consensus map was first constructed using equal weights and then again using the population sizes as weights because linkage maps based on more progeny have better resolution. For each chromosome, the best consensus map was chosen based on two criteria: (1) minimizing the average root mean-squared error (RMSE), and (2) achieving a total map length comparable to the mean of the linkage maps (Table S6).

Six markers were present in two different linkage groups and were curated manually. The duplicates for markers 1_0716, 2_1055, 1_0349, and 2_0029 were deleted based on flow-sorting results or synteny in the same manner as Muñoz-Amatriaín et al. [30]. The conflicting chromosome assignments for markers 2_0207 (4H and 7H) and 2_0883 (4H and 5H) arose from the inclusion of the iSelect MxB map and thus were not present in the previous consensus map. Both markers were arbitrarily assigned to 4H to agree with the previous consensus genetic map [30].

### Population Structure and Genetic Differentiation Analyses

The software package STRUCTURE v.2.3.4 [31] was used to infer the population structure of the NSGC barley core under an admixture model. SNPs with minor allele frequencies (MAF) <0.01 were excluded from the analysis. A subset of 1,719 SNPs representing one marker per locus was then chosen to reduce running time. STRUCTURE was run 5 times for each hypothetical number of subpopulations (*k*) between 1 and 10, with a burn-in period of 25,000 and 25,000 Monte Carlo Markov Chain (MCMC) iterations. LnP(D) values were plotted and Δ*k* values were calculated according to Evanno et al. [33] to estimate the optimum number of subpopulations. After estimating *k*, a new run using a burn-in period of 100,000 and 100,000 MCMC was used to assign accessions to subpopulations based on a membership probability greater than 0.80. Those accessions with a membership probability lower than 0.80 were assigned to an ‘admixed’ group. Principal Component Analysis (PCA) was also conducted in TASSEL v. 3.0 ([32]; Available: http://www.maizegenetics.net) on the same dataset.

Non-admixed accessions from these subpopulations were used to study the population genetic differentiation *Φ*_PT_ for each SNP. We used AMOVA results generated in the GenAlEX 6.5 with 1,000 permutations to estimate *Φ*_PT_ as
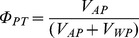
where V_AP_ is the variance among subpopulations and V_WP_ is the variance within subpopulations.

### Linkage Disequilibrium Analysis

TASSEL 3.0 ([32]; Available: http://www.maizegenetics.net. Accessed Jan 2014) was used to calculate the linkage disequilibrium (LD) parameter *r^2^* and corresponding *p*-values (two-sided Fisher’s exact test). For the calculation of LD, markers with minor allele frequency (MAF) below 0.05 and individuals with a percentage of admixture above 80% were excluded. The *r^2^* values were calculated for each chromosome for the different subpopulations and plotted against genetic distance (cM). A hundred thousand pairwise *r^2^* were calculated between randomly selected and physically unlinked markers. The distribution of those *r^2^* values was power transformed to approach normality and the parametric 99^th^ percentile of the distribution was used as a threshold to consider that LD was likely caused by genetic linkage.

A logistic regression model was used to investigate the relationship between any two markers (binary variables) as a measurement of LD. The analysis was done using SAS v9.3 PROC LOGISTIC (SAS Institute, Cary NC, USA). The advantage of this analysis over other traditional measurements of LD - such as *r^2^* - is that the logistic model allows the use of co-variables in the analysis. In our case, we included the first eleven principal components as co-variables. The significance of the regression is, therefore, a direct measurement of LD without the confounding effect of population structure [63]. The logistic model for a given response marker (M_0_) was evaluated using adjacent markers, one at a time, as regressors. To assess the variation of p-values as an indirect measure of LD decay at the position M_0_, we used regressors located at two intervals, between 1 and 2 cM and between 4 and 5 cM from M_0_.

### Genome-wide Association Analysis

GWAS was performed on the iCore using the *Q* + *K* method implemented in TASSEL v. 3.0 as a mixed linear model (MLM) function [71]. Population structure (*Q* matrix) was accounted for using the result of STRUCTURE for *k* = 5, and relatedness of accessions was corrected using a kinship matrix (*K* matrix) generated in TASSEL using SNPs with MAF>0.01. A false discovery rate (FDR; [80]) was used for multiple testing correction of the GWAS results in SAS v9.3 using the MULTTEST procedure. The -log_10_ of the adjusted *p*-values (*q*-values) were plotted against the consensus genetic position on each chromosome.

Most of the phenotypic data used for GWAS analyses were obtained from evaluations of the NSGC Barley Core germplasm found at GRIN (Germplasm Resources Information Network system. Available: www.ars-grin.gov/npgs. Accessed Jan 2014) and are shown in Table S2. Heading data were collected at Corvallis (Oregon) where the spring lines (n = 2051) were planted in the fall of 2011 and winter lines (n = 374) in the winter of 2013. Both experiments were analyzed independently. Missing ‘spike row type’ data in GRIN were completed using the data from the Corvallis experiment, where the trait was scored as ‘2-row’ or ‘6-row’. The two data sets were in agreement for all but 25 accessions, in which case we used the data obtained from the field trials at Corvallis, OR (Table S2).

### Mini-core Set Development

To select a subset of accessions that maximize the diversity of the collection, all the accessions of the collection (*n*) were sorted according to their general contribution to the average polymorphic information content (PIC) of 4,558 mapped SNPs.
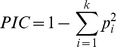

*p_i_* is the frequency of the *i* ^th^ allele *k* is the No. of alleles.

In a step wise process, all the accessions were individually removed from the dataset and the average PIC was re-calculated. The accession with the lowest contribution to the PIC (the one whose removal most increased the average PIC of the collection) was removed from the next step analysis that starts with *n-1* accessions. Sorting the accessions in this way makes it likely that a subset of lines taken from the top part of the list is the subset that represents the allelic diversity of the whole collection. The calculations were done using a Visual Basic macro built *ad hoc* in MS Excel 2013, available at: http://barleyworld.org/breeding-genetics/analysis (accessed 2014 March 24).

## Supporting Information

Figure S1 **Exploration of the optimal number of genetic subpopulations (** ***k*** **) in the iCore.** (A) Log probability of the data (LnP(D)) for each *k* between 1 and 10. (B) *Δk* values calculated as proposed by Evanno et al. [33] as a function of *k.*
(TIF)

Figure S2 **Principal Component Analysis (PCA) of the NSGC Barley iCore.** The first plot shows the proportion of variance explained by each PC, and the next three plots represent the first four PCs. Accessions are colored by the result of STRUCTURE for *k* = 5.
(TIF)

Figure S3 **Linkage disequilibrium (** ***r*** **^2^) decay over genetic distance (cM) for the seven barley chromosomes.** Significance thresholds are represented as horizontal lines.
(TIF)

Table S1 **Potential duplicates in the NSGC Core.** Each set of accessions represents potential duplicates based on the SNP information from iSelect genotyping. Accessions marked in yellow were kept, while the rest were discarded.
(XLSX)

Table S2 **Information on the 1,860 accessions belonging to the iCore.** For each accession, phenotypic and geographic information have been added when available, as well as the subpopulation each accession belongs to (1 to 5) and the proportion of each of the five subpopulations (P1–P5). ‘AD’ indicates admixed individuals.
(XLSX)

Table S3 **iSelect consensus genetic map.** Two versions of the map are presented: iSelect markers only and all SNPs.
(XLSX)

Table S4 **GWAS results of ‘hull cover’, ‘spike row number’, and ‘heading date’.**
(XLSX)

Table S5 **iCore accessions ranked by their contribution to the average Polymorphism Information Content (PIC) value of the whole set.** The reported PIC value of an accession with rank *n* correspond to the average PIC value of the set with ranks 1 to *n*-1. The spring 2- and 6-row accessions belonging to the iCore are also sorted by their contribution to the diversity of the corresponding group.
(XLSX)

Table S6 **Effect of maximum interval size on total map length and average root mean-squared error (RMSE) between linkage maps and consensus map.**
(XLSX)
