# Supplementary figures and images for: The USDA Barley Core Collection: Genetic Diversity, Population Structure, and Potential for Genome-Wide Association Studies
Source: PLoS One. 2014 Apr 14;9(4):e94688. doi: 10.1371/journal.pone.0094688 (PMC3986206; doi:10.1371/journal.pone.0094688)

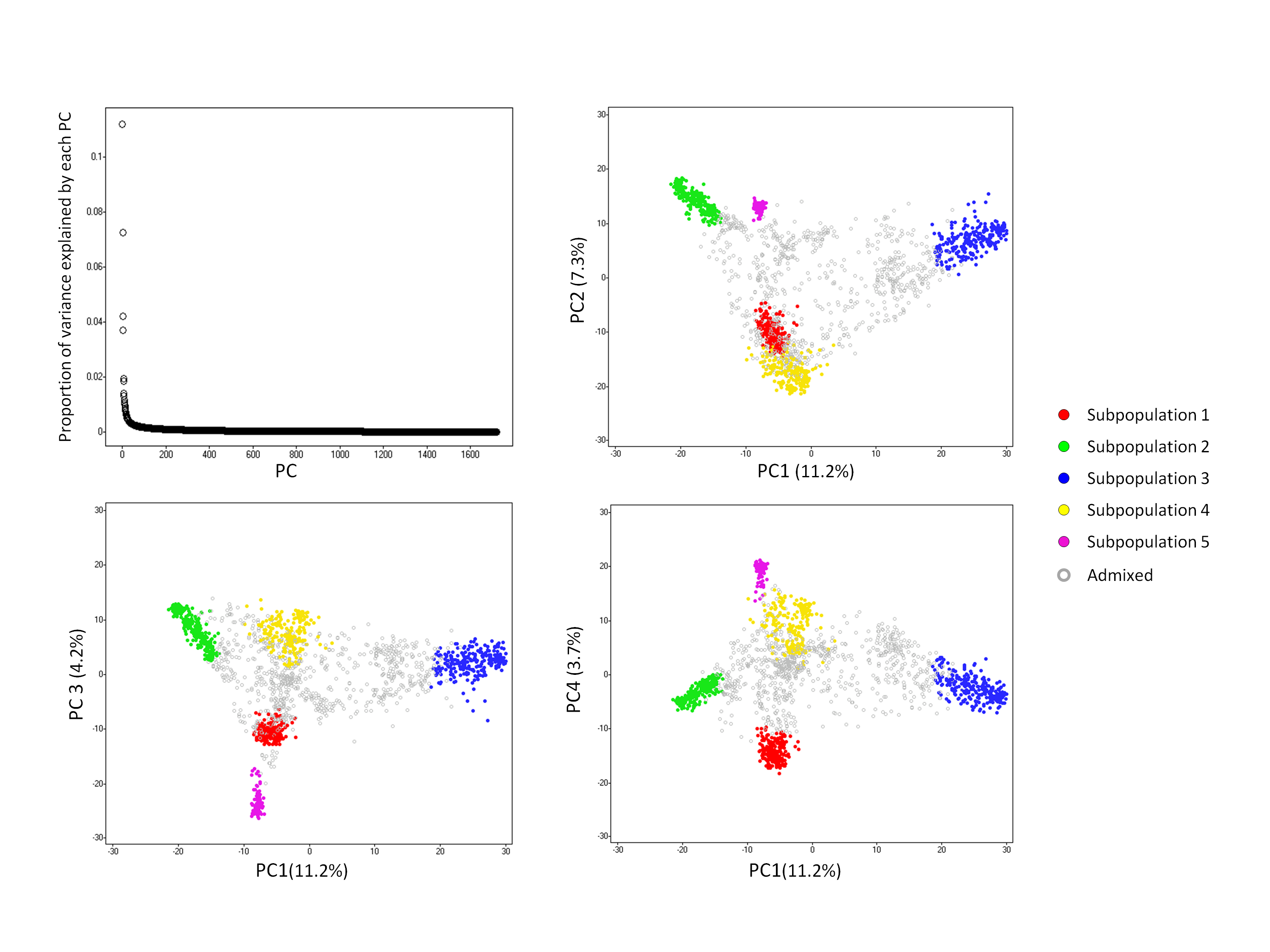

Supplement: Figure S2 — Principal Component Analysis (PCA) of the NSGC Barley iCore. The first plot shows the proportion of variance explained by each PC, and the next three plots represent the first four PCs. Accessions are colored by the result of STRUCTURE for k = 5. (TIF) [file pone.0094688.s002.tif]

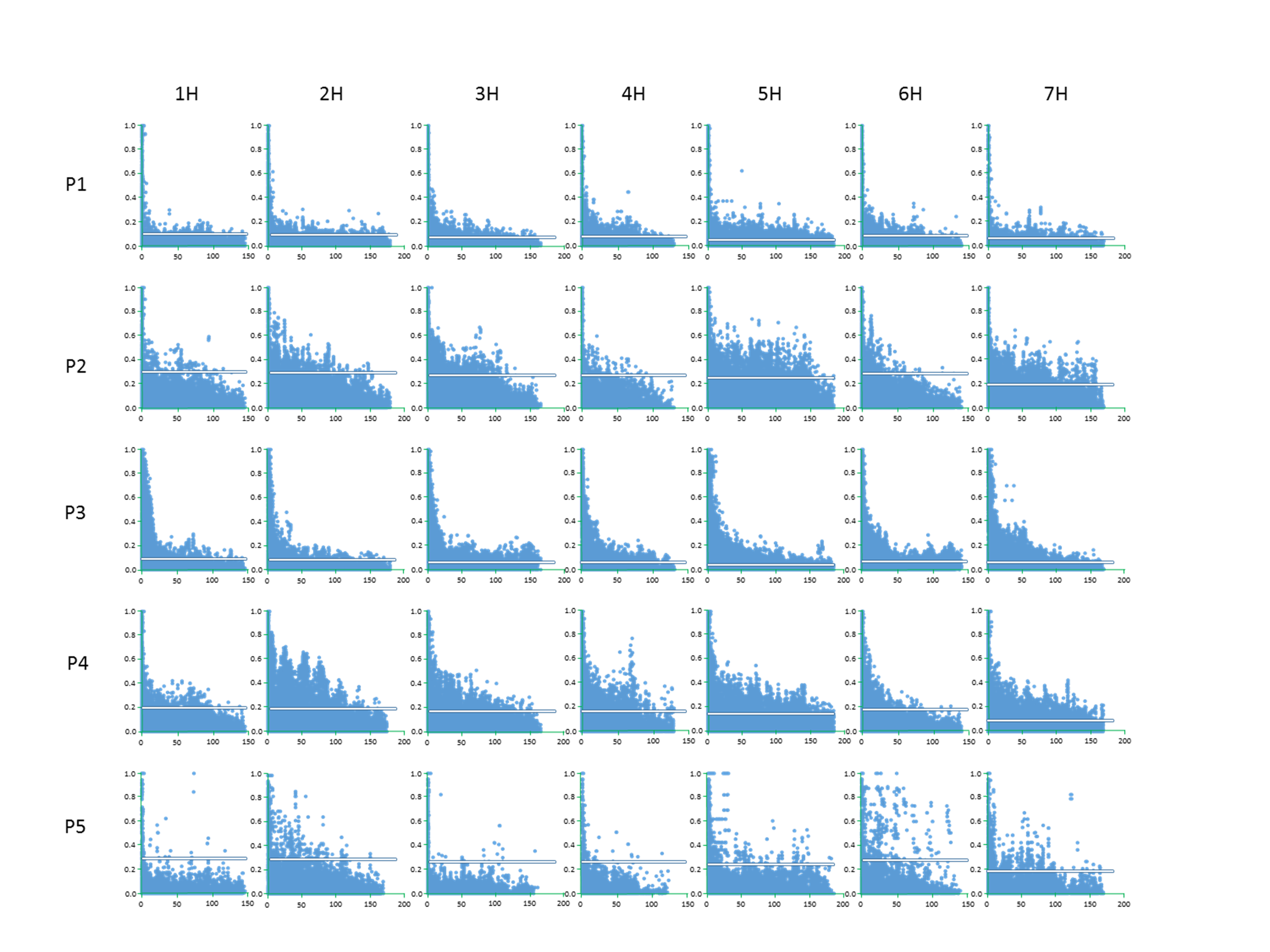

Supplement: Figure S3 — Linkage disequilibrium ( r 2) decay over genetic distance (cM) for the seven barley chromosomes. Significance thresholds are represented as horizontal lines. (TIF) [file pone.0094688.s003.tif]
